# Low Temperature Characteristics of Ge-on-Si Waveguide Photodetectors: A Combined Simulation and Experimental Study

**DOI:** 10.3390/mi16050542

**Published:** 2025-04-30

**Authors:** Jingchuan Liu, Zhenyu Li, Xiaofei Liu, Wentao Yan, Xingyan Zhao, Shaonan Zheng, Yang Qiu, Qize Zhong, Yuan Dong, Ting Hu

**Affiliations:** 1 School of Microelectronics, Shanghai University, Shanghai 201800, China; liujingchuan@shu.edu.cn (J.L.);; 2 Shanghai Collaborative Innovation Center of Intelligent Sensing Chip Technology, Shanghai University, Shanghai 201800, China; 3 Linkstar Microelectronics PTE. Ltd., Ascent 2 Science Park Drive, #01-08, Singapore 118222, Singapore

**Keywords:** Ge-on-Si photodetectors, short-wave infrared, absorption coefficients, low temperature, TCAD

## Abstract

Benefiting from the progress of the germanium (Ge) epitaxy process on silicon (Si) substrates, waveguide-integrated Ge-on-Si photodetectors (PDs) have demonstrated decent performances in short-wave infrared (SWIR) detection. By lowering the operating temperature, theses PDs can meet the stringent signal-to-noise requirements for high-sensitivity detection. We systematically investigated the dark current characteristics and optical response in the 1500–1600 nm wavelength range of the waveguide-integrated Ge-on-Si PDs operated at low temperatures (200 K to 300 K). Under a −3 V bias, the PD exhibits a room-temperature dark current of 4.62 nA and a responsivity of 0.87 A/W at 1550 nm. When the temperature was reduced to 200 K, the dark current decreased to 93.69 pA, and the responsivity dropped to 0.34 A/W. Using finite-difference time-domain (FDTD) and technology computer-aided design (TCAD) simulations, we extracted the absorption coefficients of epitaxial Ge on Si at low temperatures. At room temperature, the absorption coefficient at the wavelength of 1550 nm was approximately 1100 cm^−1^, while at 200 K, the absorption coefficient decreased to 248 cm^−1^. The outcomes of this work pave the way for the high-performance low-temperature Si photonic systems in the future.

## 1. Introduction

Compared to traditional electronic circuits, photonic integrated circuits (PICs) utilize light as the information carrier, which increases bandwidth capacity and facilitates fast transmission of large amounts of data [1,2,3,4]. In the short-wave infrared spectrum, silicon photonic integrated circuits (SiPICs) offer a compelling combination of low cost, complementary metal-oxide-semiconductor (CMOS)-compatible fabrication [5], ease of integration with electronic circuits [6], and reduced optical losses [7], making them a superior choice over platforms such as indium phosphide (InP), lithium niobate (LN), and so on [8]. A wide array of mature silicon photonic devices [9], such as the grating coupler (GC) [10], multimode interferometer (MMI) [11], microring resonator (MRR) [12], and directional coupler (DC) [13], have been extensively deployed in various fields, including optical communication, sensing, medical diagnostic, imaging and light detection, and ranging (LiDAR).

A photodetector (PD) is one of the essential components in PICs, which converts optical signals into electrical signals to realize the detection and processing of optical information. It operates based on the internal photoelectric effect, where incident photons with energy exceeding the bandgap of the semiconductor material are absorbed, generating electron-hole pairs. These carriers are then separated under an applied electric field, resulting in a measurable photocurrent. However, Si, with its large bandgap of 1.12 eV, is incapable of detecting light with wavelengths greater than 1.1 μm, which significantly limits the development of Si-based PD for infrared detection applications [14]. Fortunately, germanium (Ge), a material from the same group as Si, possesses a narrower bandgap of 0.66 eV, allowing its absorption spectrum to cover the wavelength range from 1300 nm to 1600 nm [14]. Although Ge is an indirect bandgap material, the lattice mismatch between Si and Ge (approximately 4.2%) induces strain when Ge is epitaxially grown on a Si substrate [15]. This strain enhances its absorption coefficient in the C-band to approximately five times that of bulk Ge [16]. As a result, germanium-on-silicon (Ge-on-Si) PD can achieve a responsivity exceeding 0.8 A/W at a wavelength of 1550 nm, rivaling that of indium gallium arsenide (InGaAs) PD [17,18].

In key technological domains such as infrared detection, imaging, and LiDAR systems, high-sensitivity PDs serve as crucial components. However, the inherent lattice mismatch between Si and Ge results in relatively large dark current noise in Ge-on-Si PDs, limiting their application in high-sensitivity detection. Fortunately, dark current exhibits a strong temperature dependence, decreasing substantially with reduced operating temperatures. There are different approaches to realize the low-temperature operation condition for a Ge-on-Si PD. Compared to vapor-compression refrigeration, thermoelectric coolers (TECs) offer several advantages, including the absence of moving parts, a compact form factor, noiseless performance, and precise temperature control capabilities [19]. Packaging the PD with a micro-TEC still maintains the benefits of compactness and high integration. Currently, three-stage TECs can achieve a temperature range from 200 K to 300 K when the hot side is maintained at room temperature [20]. Consequently, investigating the low-temperature characteristics of Ge-on-Si PDs at the temperature range that can be realized using TECs is of paramount importance for advancing the development of high-sensitivity Si photonic detector systems.

The investigation of low-temperature electrical models for Si and Ge has reached a significant level of maturity, encompassing comprehensive studies on band structure models, mobility models, and carrier generation-recombination models [21,22,23,24,25,26,27]. Furthermore, the low-temperature absorption coefficient of epitaxial Ge films on Si plays a pivotal role in evaluating and optimizing the optical response of Ge-on-Si PDs at low temperature. Previous research by J. Xu et al. [21] characterized the refractive index of polycrystalline Ge film at wavelengths between 1529 nm and 1560 nm within the temperature range from 253 K to 283 K. V. In addition, M. Závěctová et al. [28]. investigated the absorption characteristics of epitaxial Ge on glass from 1000 nm to 2000 nm at both 300 K and 80 K. The 1500–1600 nm spectral band is particularly significant for numerous applications, including long-haul optical communications, LiDAR systems for autonomous vehicles, precision gas sensing, and biomedical imaging and diagnostics. Recent advances in micro-TEC and Ge-on-Si PD packaging technologies now enable low-temperature operation (200–300 K), offering enhanced signal-to-noise ratios and improved detection sensitivity. However, a critical knowledge gap remains regarding the temperature-dependent absorption characteristics of epitaxial Ge films on Si, specifically in the wavelength range from 1500 nm to 1600 nm and temperature range from 200 K to 300 K.

In this work, we experimentally characterized the dark current, photocurrent, and responsivity of waveguide-integrated vertical PIN (VPIN) Ge-on-Si PD operating at low temperature (200 K to 300 K) and wavelengths between 1500 nm and 1600 nm. Furthermore, we developed simulation models using finite-difference time-domain (FDTD) and technology computer-aided design (TCAD) tools to extract the low-temperature absorption coefficient of Ge across a wavelength range from 1500 nm to 1600 nm. This result is particularly relevant for optimizing next-generation, low-temperature-operated photonic systems requiring high sensitivity.

## 2. Device Structure

**Figure 1:**
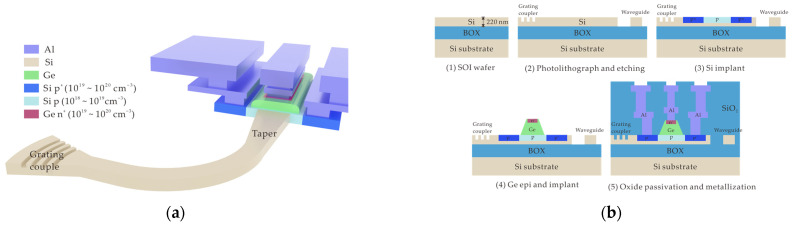
(**a**) The 3D schematic of the waveguide Ge-on-Si VPIN PD. (**b**) The diagram of the fabrication process flow.

**Figure 2:**
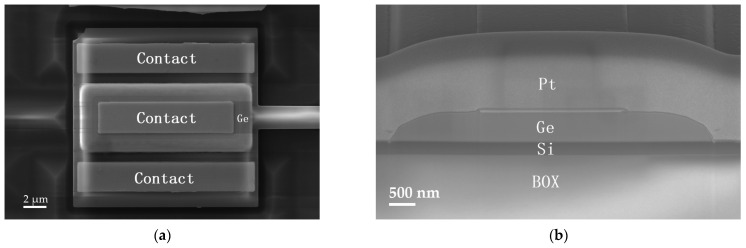
(**a**) Top-view SEM image of the PD. (**b**) Cross-sectional SEM image of the PD. The cross-section was realized using the FIB milling process.

Figure 1a,b illustrate the three-dimensional (3D) schematic of the PD and fabrication process flow, respectively. The PD was fabricated on a 220 nm silicon-on-insulator (SOI) wafer, where the grating coupler, taper, and waveguide were realized through photolithography and etching processes. Subsequently, two rounds of boron ion implantation were performed in the PD region: the first to form the p region and the second to create the p^+^ contact region. On the p region, approximately 500 nm-thick intrinsic Ge was selectively grown using the epitaxial technique, followed by phosphorus ion implantation into the Ge layer to form the n^+^ region. Finally, the PD was passivated with silicon dioxide, and contact vias were opened to form aluminum metal electrodes. Light is coupled into the chip through the grating coupler and guided into the PD via a 100 μm-long taper. Figure 2a,b displays the top-view and cross-sectional scanning electron microscopy (SEM) images of the PD, respectively. These images provide detailed structural information about the Ge layer. Based on the precise structural dimensions, we constructed a simulation model to perform numerical simulations, and the results were compared with experimental data.

## 3. Results and Discussion

### 3.1. Dark Current Characteristics and the Simulation Model

The leakage current in PDs can be categorized into two distinct components: bulk leakage current and surface leakage current. The bulk leakage current primarily originates from the generation of minority carriers within the Ge layer, with its recombination rate governed by the Shockley–Read–Hall (SRH) model [29]:

(1) $$R_{SRH}=\frac{pn-{n_{ie}}^{2}}{\tau_{p}\left[n+n_{ie}\exp\left(\frac{E_{trap}}{kT}\right)\right]+\tau_{n}\left[p+n_{ie}\exp\left(-\frac{E_{trap}}{kT}\right)\right]}$$

where $n_{ie}$ is the intrinsic carrier concentration, $E_{trap}$ is the difference between the trap energy level and the intrinsic Fermi level, and $\tau_{p}$ and $\tau_{n}$ are the electron and hole lifetimes in bulk. The carrier lifetime exhibits significant dependence on multiple physical parameters, including impurity concentration, operational temperature, and electric field, which can be modeled using the following [24]:

(2) $$\tau =\left[\tau_{min}+\frac{\tau_{max}-\tau_{min}}{1+{\left(N_{total}/N_{ref}\right)}^{\gamma }}\right]\frac{f(T)}{1+\Gamma (E)}$$

where $N_{total}$ is the total dopant concentration, and f(T) and Γ(E) represent the temperature-dependent term and the field-effect enhancement term, respectively. The surface leakage current predominantly originates from the generation of minority carriers at the Ge/SiO_2_ interface, which can be quantitatively characterized by the surface recombination velocity as follows [30,31]:

(3) $$\tau_{eff}=\frac{\tau_{0}}{1+s\tau_{0}C_{surf}}$$

where $\tau_{0}$ is the bulk carrier lifetime, s is the recombination velocity, and $C_{surf}$ is an interfacial coupling coefficient, calculated locally based on the geometry of the computational grid. It is evident that the leakage current in PDs is predominantly influenced by several critical factors, including environmental temperature, material properties (including bandgap energy, defects, and impurity concentrations), applied electric field strength, and surface recombination.

In TCAD simulations, in addition to the aforementioned SRH model, the carrier mobility model plays a pivotal role in determining the current–voltage characteristics of PDs. Mobility modeling is normally divided into low-field behavior and high-field behavior. For Si and Ge, the Arora model [32] and the Klaassen model [23,33] are used, respectively, to describe low-field mobility, which is dependent on phonon and impurity scattering. At high electric fields, the mean drift velocity of carriers no longer increases linearly with the increasing electric field. Instead, it saturates at a specific value $\nu_{sat}$, which is dependent on the lattice temperature [23]. To describe the transition between low-field and high-field behavior, the Caughey and Thomas expression is given in reference [34].

**Figure 3:**
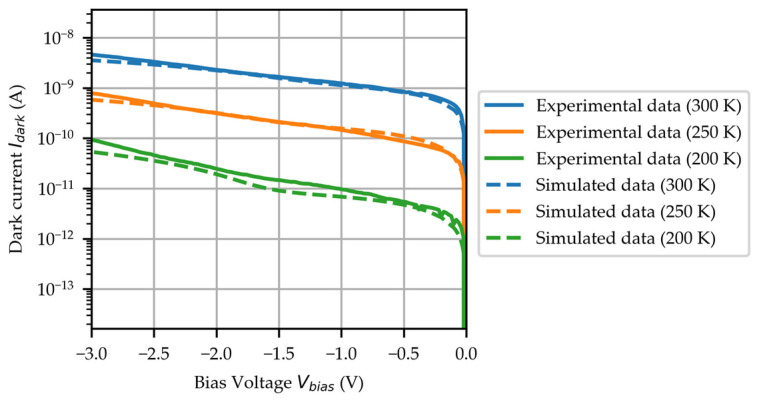
The $I_{dark}-V_{bias}$ curves as the temperature ranges from 200 K to 300 K, including the experimental measurements (solid lines) and the simulation results (dashed lines).

The dark current–voltage ($I_{dark}-V_{bias}$) characteristics of the PD within the temperature range from 200 K to 300 K and were measured using a helium-cooling probe station with a Keithley 4200A-SCS parameter analyzer (Keithley, Beaverton, OR, USA). The dark current $I_{dark}$, as a function of bias voltage $V_{bias}$, from 0 V to −3 V at different temperatures, is illustrated in Figure 3, where the solid lines represent the experimental measurements and the dashed lines correspond to the TCAD simulation. At room temperature (300 K), the dark current of the PD is 4.62 nA at the $V_{bias}=-3\;V$, while at 200 K, the dark current significantly decreases to only 93.63 pA, representing a 50-fold reduction. This substantial decrease in dark current can be attributed to several temperature-dependent phenomena: the reduction in thermally excited carriers, decreased carrier recombination rates, weakened tunneling effects, and the diminished influence of material defects. These combined effects lead to a remarkable suppression of dark current noise, which is crucial for enhancing the PD’s performance in high-sensitivity detection applications.

### 3.2. Photo-Response Characteristics and the Simulation Model

When photons with energy equal to or greater than the bandgap energy of the semiconductor material are incident on Ge, they are absorbed, promoting electrons from the valence band to the conduction band and thereby generating electron–hole pairs, known as photogenerated carriers. For non-magnetic materials in a time-harmonic electromagnetic field, based on Maxwell’s equations and the Poynting theorem, the absorption per unit volume is given as follows:

(4) Pabs=12ωE→2Im(ε)=ε0ωnkE→2

where Im denotes the imaginary part, $\vec{E}$ is the complex amplitude of the electric field, ω is the angular frequency, *ε* represents the permittivity of the material, $\varepsilon_{0}$ represents the permittivity of free space, and n and k denote the refractive index and extinction coefficient of the material, respectively. Finally, the photogeneration rate G in the Ge region is determined by dividing the absorption per unit volume by the energy of a single photon:

(5) $$G=\frac{P_{abs}}{ћ\omega }=\frac{\varepsilon_{0}nk}{ћ}{\left|\vec{E}\right|}^{2}$$

where ћ is the reduced Planck constant. The photocurrent of the device can be determined by substituting the G into the carrier continuity equations,

(6) $$\begin{array}{l} \frac{\partial n}{\partial t}=\frac{1}{q}\nabla \cdot {\vec{J}}_{n}+G-R\\ \frac{\partial p}{\partial t}=\frac{1}{q}\nabla \cdot {\vec{J}}_{p}+G-R \end{array}$$

where *q* is the elementary charge, ${\vec{J}}_{n}$ and ${\vec{J}}_{p}$ represent the electron current density and hole current density, respectively, and *R* is the recombination rate, which can be obtained using Equation (1). Briefly, the photocurrent in the intrinsic region can be approximated by integrating the *G* over its three-dimensional volume as follows:

(7) $$I_{\mathrm{ph},\mathrm{intrinsic}}=q{∭}_{i\text{-}\mathrm{region}}\eta \cdot Gdxdydz$$

where *η* represents the efficiency with which photogenerated carriers are collected into the current.

**Figure 4:**
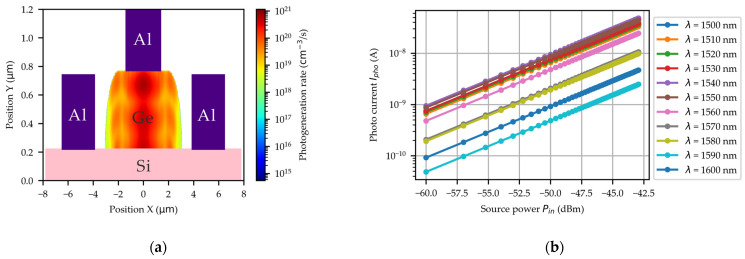
(**a**) The simulated cross-sectional profile of the photogeneration rate with the λ=1550 nm. (**b**) The simulated $I_{pho}-P_{in}$ curves for the wavelength ranging from 1500 nm to 1600 nm at room temperature.

In FDTD simulations, the temperature-dependent refractive index parameters of materials play a critical role in determining electromagnetic wave propagation characteristics at different temperatures. The optical constants for Si, Ge, and SiO_2_ were adopted from reference [35,36,37]. The TE-mode optical input was launched into a 500 nm × 220 nm Si strip waveguide, while the electromagnetic field distribution within the Ge region was recorded. The photogeneration rate was subsequently calculated using Equation (5). Figure 4a presents the photogeneration rate cross-sectional profile within the Ge region, calculated using the FDTD method under 1550 nm wavelength illumination with an incident power of 1 nW. Based on the spatial distribution of photogeneration, the photocurrent variation as a function of optical power can be simulated, as illustrated in Figure 4b. The photocurrent increases linearly with the power of the light source. When the wavelength of the light source is greater than 1550 nm, there is a significant decrease in photocurrent. By calculating the slope of these curves, the responsivity of the PD can be obtained.

**Figure 5:**
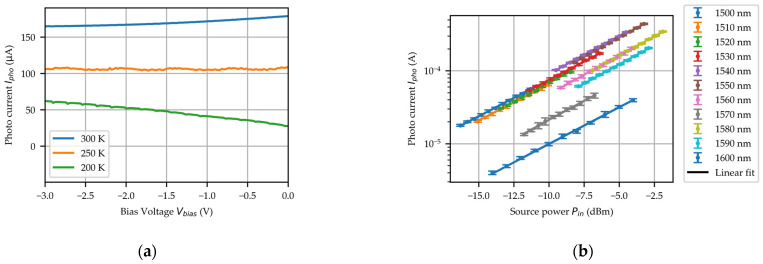
(**a**) The measured $I_{pho}-V_{bias}$ curves of PD at different temperatures illuminated with the λ=1550 nm and the $P_{in}=-7.32\;\mathrm{dBm}$. (**b**) The measured $I_{pho}-P_{in}$ curve of the PD at room temperature.

The optical response of the PD was systematically characterized across the 1500–1600 nm wavelength range under controlled temperature conditions (200–300 K) using a helium-cooling probe station and a Keysight N7778C tunable laser source. Figure 5a presents the photocurrent–voltage ($I_{pho}-V_{bias}$) characteristics of the PD under 1550 nm wavelength illumination with an incident optical power of −7.32 dBm at three operating temperatures: 300 K, 250 K, and 200 K. The results demonstrate a clear temperature dependence of the photocurrent, showing an obvious reduction with decreasing temperature. At room temperature, the PD exhibits a photocurrent of 167.8 μA, corresponding to a responsivity of 0.87 A/W. When the temperature is reduced to 200 K, the photocurrent decreases to 57.7 μA, yielding a reduced responsivity of 0.34 A/W. This 2.6-fold decrease in responsivity can be attributed mainly to the decreased absorption coefficient resulting from the bandgap energy of Ge at low temperatures. Figure 5b presents the photocurrent versus optical power ($I_{pho}-P_{in}$) characteristics of the PD under −3 V bias at room temperature for various illumination wavelengths. The photocurrent exhibits a linear increase with optical power at each specific wavelength. Through linear regression analysis, the responsivity at each wavelength was quantitatively determined. At room temperature, the PD maintains responsivity exceeding 0.65 A/W across the 1500–1550 nm spectral range. However, a sharp responsivity decline is observed beyond 1560 nm, reaching only 0.087 A/W at 1600 nm. This abrupt decrease correlates with Ge’s fundamental material properties, as the direct bandgap energy of Ge is 0.8 eV.

**Figure 6:**
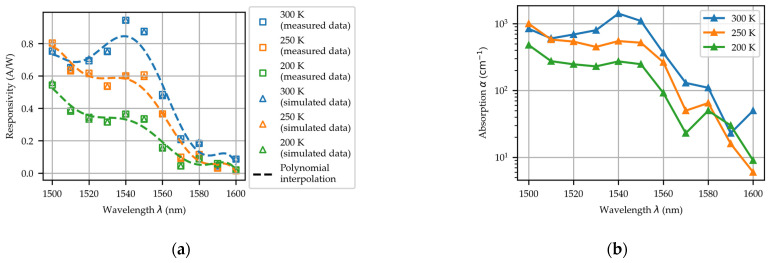
(**a**) The experimental (squares) and simulated (triangles) responsivity of the PD at different wavelengths and temperatures. (**b**) The absorption coefficient of Ge at different wavelengths and temperatures was extracted from the simulation model.

By systematically varying the absorption coefficient of Ge in models, we numerically simulated the PD’s responsivity across a 1500–1600 nm wavelength range and 200–300 K temperature range using the FDTD method and TCAD simulations. Figure 6a presents the measured and simulated responsivity data across different wavelengths and temperatures. The dashed line represents a polynomial interpolation of the experimental responsivity data within the 1500–1600 nm wavelength range, illustrating the trend of responsivity variation with wavelength. It is observed that the simulation model could provide a decent match with the experimental results. By extracting the absorption coefficient parameters from this simulation model, the wavelength- and temperature-dependent absorption coefficients of Ge were obtained, as shown in Figure 6b. At room temperature, the absorption coefficient of Ge epitaxially grown on Si is around 1100 cm^−1^ at a wavelength of 1550 nm, while at λ = 1600 nm, the absorption coefficient becomes only around 50 cm^−1^. As the temperature decreases, the absorption coefficient of Ge drops significantly, reaching only 248 cm^−1^ at 200 K, and λ=1550 nm. The absorption coefficient of Ge exhibits strong dependence on both temperature and wavelength. As temperature decreases, the bandgap of Ge widens, leading to a corresponding reduction in its absorption coefficient. When the photon energy falls below the direct bandgap threshold of Ge (~0.8 eV), the absorption coefficient undergoes an abrupt decrease. The low-temperature absorption coefficients of Ge obtained in this work provide crucial material parameters for designing low-temperature-operated Ge-on-Si PDs with an optimized optical response in the 1500–1600 nm window. These findings lay a solid foundation for the performance optimization of integrated micro-TEC/PD modules.

## 4. Conclusions

In this study, we investigated the dark current characteristics and optical response of a waveguide-integrated vertical PIN (VPIN) Ge-on-Si photodetector at low temperatures (200 K to 300 K) and a wavelength range from 1500 nm to 1600 nm. For a reverse bias of 3 V, the dark current at room temperature was measured to be 4.62 nA, which decreased to 93.63 pA at 200 K, representing a reduction by a factor of approximately 50. Similarly, the responsivity with a wavelength of 1550 nm decreased from 0.87 A/W at room temperature to 0.34 A/W at 200 K. These complex temperature-dependent variations are influenced by factors such as the quality of the Ge epitaxial layer, minority carrier generation in the space charge region, and changes in the band structure.

Based on the PD’s geometrical structure and material parameters, we developed an FDTD model and an electrical transport model to simulate its dark current and optical response characteristics. By comparing the simulation results with experimental data, the absorption coefficients and extinction coefficients of epitaxial Ge on Si at a temperature range from 200 K to 300 K and a wavelength range from 1500 nm to 1600 nm were extracted. At room temperature, the absorption coefficient of epitaxial Ge at 1550 nm is approximately 1100 cm^−1^, while at 200 K, the absorption coefficient decreased to around 248 cm^−1^. Traditionally, the absorption coefficient of Ge thin film is usually obtained using ellipsometry. However, it is challenging to perform ellipsometry for the micrometer-scale (or even sub-micrometer size) Ge structures obtained using the selective epitaxy process in the current Si photonics foundry platform, whereas our method provides a feasible approach to obtain the absorption coefficient of the selectively grown Ge at various temperature conditions. The results of this work provide insights into the temperature-dependent optical characteristics of the epitaxial Ge films on Si and their impacts on the performance of PDs, offering a foundation for optimizing the design of high-sensitivity PDs in PICs operated at low temperature.

## Data Availability

The data are available on request from the authors.
